# Validation of Axial Juvenile Spondyloarthropathy Criteria in Turkish Juvenile Spondyloarthropathy Patients

**DOI:** 10.3390/diagnostics15121498

**Published:** 2025-06-12

**Authors:** Dilara Unal, Cansu Ayten Tatar, Erdal Sag, Yagmur Bayindir, Emil Aliyev, Veysel Cam, Hulya Ercan Emreol, Mehmet Orhan Erkan, Ozge Basaran, Yelda Bilginer, Seza Ozen

**Affiliations:** Department of Pediatric Rheumatology, Hacettepe University, 06100 Ankara, Turkeyyagmur.bayindir@hacettepe.edu.tr (Y.B.); hulyaercanemreol@hacettepe.edu.tr (H.E.E.);

**Keywords:** axial spondyloarthritis, classification, arthritis, spondyloarthropathies, enthesitis

## Abstract

**Background:** Juvenile spondyloarthritis (JSpA) is a heterogeneous group of diseases. An international consensus group developed the axial juvenile SpA (AxJSpA) classification criteria for this purpose, defining a homogeneous group of patients diagnosed with jSpA and experiencing axial symptoms before the age of 18 years. **Aim:** To validate this new set of criteria in our pediatric SpA patients. **Methods**: This study was held in the Hacettepe University Department of Pediatric Rheumatology. Juvenile SpA patients suspected of axial disease diagnosed and followed at the same center between 2005 and 2024 were included. Patients who had other etiologies for axial symptoms, including chronic nonbacterial osteomyelitis, mechanical back pain–overuse injuries, amplified pain/growing pains, and SAPHO syndrome (synovitis, acne, pustulosis, hyperostosis, and osteitis) served as the control group. **Results:** In total, 123 JSpA patients and 74 controls were included in this study. The sensitivity/specificity of the new criteria were 61%/77% with an area under curve value of 0.75 (95% CI: 0.68–0.83) in our cohort. Among different criteria sets, European Spondyloarthropathy Study Group (ESSG) criteria were the most sensitive (sensitivity/specificity 91%/68%), and ASAS peripheral criteria (Assessment of SpondyloArthritis International Society) were the most specific (sensitivity/specificity 67%/84%) in our cohort when compared to ASAS axial criteria (sensitivity/specificity 74%/65%), ILAR (International League of Associations for Rheumatology) (sensitivity/specificity 85%/81%), and ILAR + SI (sacroiliitis) (sensitivity/specificity 67%/74%) criteria. **Conclusions:** The area under the curve of the new AxJSpA criteria was similar to that of the original report; however, both sensitivity and specificity were lower in our cohort, possibly due to factors like earlier disease presentation and a lower prevalence of chronic structural changes on MRI.

## 1. Introduction

Juvenile spondyloarthritis (JSpA) encompasses a group of heterogeneous diseases characterized by peripheral arthritis, axial involvement, enthesitis, acute anterior uveitis, psoriasis, inflammatory bowel disease (IBD), and HLA-B27 positivity. The prevalence of JSpA can vary significantly, ranging from 10 to 30% of all juvenile idiopathic arthritis (JIA) patients, depending on the geographic area [1,2,3,4]. Children with JSpA have a significant disease burden and relatively poor outcomes compared to other types of JIA [5]. Among children with JSpA [6], 10% to 20% of patients develop ankylosing spondylitis (AS) in adulthood [7]. Patients with JSpA share many similarities with adult spondyloarthritis; however, there are also significant differences between the two. Inflammatory back pain is not as prominent, and the sacroiliac and other vertebral joints are less frequently affected in juvenile disease [8]. In contrast, children often present with hip and peripheral arthritis, along with enthesitis. In pediatric cases, magnetic resonance imaging (MRI) may misinterpret certain physiological bone marrow changes as sacroiliitis [9]. A significant proportion of children with JSpA experience “silent” axial disease, which refers to axial involvement without back pain. Even in cases without reported back pain, MRI scans reveal evidence of both acute disease and chronic destructive changes [10].

Several diagnostic and/or classification criteria have been proposed for JSpA and adult-onset spondyloarthropathies. The International League of Associations for Rheumatology (ILAR) JIA classification criteria are the most commonly used pediatric criteria; however, there is ongoing debate about their performance in classifying juvenile spondyloarthropathies [11]. These classification criteria primarily categorize patients with JSpA as either enthesitis-associated arthritis or psoriatic arthritis. These criteria do not include inflammatory-bowel-disease-related arthritis, juvenile ankylosing spondylitis, or Reiter’s syndrome, which are well-defined forms of JSpA. Furthermore, the enthesitis-related arthritis category and stringent classification criteria often lead to many patients being classified as having undifferentiated arthritis [12]. On the other hand, the SpA International Society (ASAS) currently categorizes adult SpAs using two sets of criteria. One set is for patients experiencing axial involvement, while the other is for those with peripheral symptoms [13,14,15]. There are two types of axial SpA (axSpA). These are radiographic axSpA, which is characterized by the presence of clear radiographic axial disease and is known as AS, and non-radiographic axSpA [11].

Early recognition of JSpA and, especially, axial involvement is crucial for reducing long-term disease burden and preventing irreversible damage by starting earlier and more aggressive treatment. Recently, an international collaboration developed the axial juvenile SpA (AxJSpA) classification criteria, aimed at identifying juvenile patients with axial symptoms before 18 years of age, and they validated it in an independent group [16]. We designed the present study to validate the recently published AxJSpA classification criteria in a cohort of JSpA patients and to assess the effectiveness of the ASAS axial SpA criteria [13], the ILAR ERA criteria [17], the ILAR ERA definition of sacroiliitis [11], the ASAS peripheral SpA criteria [15], and the European Spondyloarthropathy Study Group (ESSG) criteria for adults [18]. We hypothesize that the new AxJSpA classification criteria will provide a valid and reliable tool for classifying juvenile spondyloarthritis with axial involvement in a pediatric cohort.

The AxJSpA criteria demonstrated moderate specificity and sensitivity in our JSpA cohort. The observed variability among the existing classification criteria highlights the diagnostic challenges in juvenile spondyloarthritis. Importantly, early detection of axial involvement is crucial for optimal disease management and timely intervention.

## 2. Materials and Methods

We retrospectively evaluated the medical records of SpA patients under 18 years of age at diagnosis and followed up for at least 6 months at the Pediatric Rheumatology Department of Hacettepe University, Ankara, between January 2005 and January 2024. We enrolled 123 SpA patients (113 ERA, 10 PsA) and validated the AxJSpA classification criteria using real patient data analysis (Supplementary Figure S1).

We included controls similar to the reference study, which had other etiologies for axial symptoms, including chronic nonbacterial osteomyelitis (CNO) (*n* = 44), mechanical back pain–overuse injuries (*n* = 18), amplified pain/growing pains (*n* = 8), and SAPHO syndrome (*n* = 4). Patients with familial Mediterranean fever (FMF) with sacroiliitis, as well as those with sacroiliitis secondary to infections, drugs, undifferentiated arthritis, or reactive arthritis, were excluded from the study. These conditions represent distinct pathophysiological entities that could mimic SpA and confound the classification process; therefore, they were excluded to maintain consistency with the original AxJSpA validation study. Demographic characteristics, clinical manifestations, laboratory findings, and the items of AxJSpA classification criteria, ASAS classification criteria for axial and peripheral SpA, ILAR ERA criteria, the ILAR ERA definition of sacroiliitis, and the European Spondyloarthropathy Study Group (ESSG) criteria for adults were also assessed (Table 1 and Table 2). Regarding the control group’s classification, an experienced pediatric rheumatologist’s expert opinion (SO) was used as the gold standard. The classification was based on a thorough review of clinical, imaging, and laboratory findings, ensuring the exclusion of conditions that could mimic axial involvement. The sensitivities and specificities of AxJSpA classification criteria, ASAS classification criteria for axial and peripheral SpA, ILAR ERA criteria, the ILAR ERA definition of sacroiliitis, and the European Spondyloarthropathy Study Group (ESSG) criteria for SpA patients were also evaluated according to the features registered at disease diagnosis. Sensitivity, specificity, and predictive values were calculated using cross-tabulation against the clinician-based diagnosis (gold standard). This study has been approved by the Hacettepe University Ethics Commission (approval number: GO 21/665). All participants provided informed consent.

## 3. Statistical Analysis

Statistical analyses were performed by using the SPSS (Statistical Package for the Social Sciences) version 29.0 package program. Descriptive statistics were presented as frequency, percentage, median, minimum (min), and maximum (max). The variables were investigated using visual (histograms, probability plots) and analytical methods (Kolmogorov–Smirnov) to determine whether they were normally distributed. The Chi-square test or Fisher’s exact test (when Chi-square test assumptions did not hold due to low expected cell counts) was used to analyze relationships between categorical variables. The Mann–Whitney *U* test was used to compare non-normally distributed continuous variables. A *p* value of less than 0.05 was considered significant. Sensitivity, specificity, and positive and negative predictive values of the AxJSpA classification criteria against the gold standard clinician’s diagnosis were calculated and used to assess the diagnostic accuracy of the AxJSpA classification criteria and compared with the ASAS axial SpA criteria [13], the ILAR ERA criteria [17], the ILAR ERA definition of sacroiliitis [11], the ASAS peripheral SpA criteria [15], and the European Spondyloarthropathy Study Group (ESSG) criteria for adults [18]. Receiver operator characteristic (ROC) analysis was used to determine a suggested cut-off probability for the AxJSpA classification criteria in our pediatric cohort.

## 4. Results

### 4.1. Demographic Features of the Patients

This study included 123 JSpA patients (26% female) and 74 non-JSpA (47.3% female) controls. The control group consisted of patients with CNO (*n* = 44), mechanical pain (*n* = 18), growth pain (*n* = 8), and SAPHO syndrome (*n* = 4). The mean age at diagnosis was 13 ± 2.8 years in the JSpA patients and 10.73 ± 3.8 years in the control group. Table 3 summarizes the patient demographics and clinical characteristics.

The disease duration at the time of diagnosis was frequently 12 weeks or more in the case and control groups at 90.2% and 78.4%, respectively (*p* = 0.021). Hips or the groin were the most common areas of localization of pain in JSpA patients (*n* = 98, 80%) and controls (*n* = 42, 56.8%) (*p* 0.001). Acute disease onset was observed in 69.9% (*n* = 86) of patients with JSpA, whereas an insidious onset pattern was more common than acute onset among individuals in the control group (33.8%, *n* = 25). Peripheral arthritis and enthesitis were more common in the case group (both *p* < 0.01). Morning stiffness was commonly more prolonged (≥30 min in 36.6%) in the JSpA patients than the control group.

### 4.2. Sensitivity and Specificity of the New AxJSpA Classification Criteria in JSpA Patients

**Figure 1:**
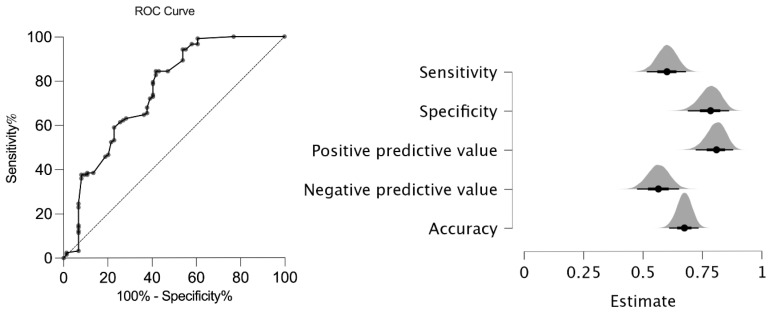
ROC analysis and performance of the new AxJSpA criteria in JSpA patients. (ROC: receiver operator curve; JspA: juvenile spondyloarthritis).

The new AxJSpA criteria were validated in our cohort, and the validity of the new classification system was compared to that of the ASAS axial SpA criteria [14], the ILAR ERA criteria [17], the ILAR ERA definition of sacroiliitis [11], the ASAS peripheral SpA criteria [15], and the European Spondyloarthropathy Study Group (ESSG) criteria [18] for adults. In our cohort, the AxJSpA criteria had a specificity of 77% and a sensitivity of 61% with an Area Under the Receiver Operating Characteristic (AUROC) curve of 0.75 (95% CI: 0.68–0.83) (Figure 1). 

Comparison with ILAR ERA criteria [17], the ILAR ERA definition of sacroiliitis [11], ASAS axial and peripheral SpA [14], and ESSG [18] criteria for adults showed that the sensitivity of the EESG criteria (92%) was the highest, while the highest specificity was that of the ASAS peripheral criteria (84%). The new AxJSpA criteria had the lowest negative predictive value (53.7%), together with a relatively high positive predictive value (81.30%) (Table 4).

The results of the sensitivity and specificity of each parameter of the new AxJSpA classification criteria are presented in Table 5.

## 5. Discussion

This is the first external validation of the new AxJSpA classification criteria in a JSpA population. Our study cohort and the original validation cohort were comparable in terms of gender distribution, age at diagnosis, and HLA-B27 positivity; however, notable differences included a higher frequency of peripheral arthritis, a lower prevalence of MRI-detected chronic structural changes, and a predominance of acute disease onset in our cohort. In contrast to the original validation cohort, our patients had a lower frequency of uveitis and inflammatory bowel disease, while the rate of enthesitis was similar between the two groups.

In our cohort, we have found a similar sensitivity (61%) but a relatively low specificity (77%) with a similar area under the curve of 0.75 (95% CI: 0.68–0.83) than those reported by Weiss et al. [16]. The original study reported a specificity of 97.5%, a sensitivity of 64.3%, and an area under the curve of 0.81 (95% CI: 0.76–0.86) in their validation cohort according to the new AxJSpA classification using the threshold score of ≥55 (out of 100). Imaging typical of sacroiliitis was regarded as a required but insufficient criterion for AxJSpA classification. Comparing our study to the original one, we found relatively low sensitivity and a low negative predictive value of 53.70%. One reason may be the lack of chronic sacroiliac lesions in our patient group. Many of our patients were evaluated early in the disease course, often at the onset of axial symptoms, allowing for the detection of active inflammatory lesions before the development of structural damage. In parallel, several factors may also account for the relatively lower specificity observed in our cohort.

In the original study, imaging evidence of juvenile SpA was required alongside clinical symptoms. However, MRI interpretation in children can be challenging due to thick cartilaginous growth plates, which may be falsely interpreted as sacroiliitis [10]. Variability in radiologist expertise and institutional criteria may further contribute to inconsistencies. Additionally, conditions like CNO can show sacroiliitis-like changes on MRI [19], and the presence of such cases in our control group may have contributed to lower specificity.

It is worth noting that while imaging evidence is necessary to surpass the classification threshold, there are cases of JSpA with imaging evidence of typical axial disease in juvenile SpA that are below the threshold. These were cases for which clinical criteria levels had a low score. Similarly, there were cases that had near maximum level scoring for each of the clinical domains but no imaging evidence typical of juvenile SpA. While 43 out of 49 patients who were false negatives had clinical features compatible with SpA, they did not have sufficient MRI findings, and 37 of these patients had peripheral arthritis, while 31 had enthesitis. Twenty-one patients did not fulfill the criteria due to the presence of acute sacroiliitis on MRI but no chronic structural lesions. Analyzing the individual parameters of the AxJSpA classification criterion reveals a sensitivity of 47.9% for structural lesions but a specificity of 78.3%. Given the classification criteria’s emphasis on specificity, it makes sense to incorporate chronic changes into the criteria. However, the lower frequency of chronic changes in our study may have contributed to the low sensitivity.

A study examining the occurrence of sacroiliitis at diagnosis in cases of JSpA revealed that fifteen children with JSpA exhibited sacroiliac arthritis according to the ILAR definition [11], although not with MRI [10]. Other investigations have indicated that the primary presentation of early illness in children is peripheral arthritis and enthesitis, rather than inflammatory back pain [20]. The early detection of axial disease has been enabled by the increased use of MRI [21,22]. Anti-TNF agents have demonstrated efficacy in clinically enhancing the signs and symptoms of the disease [23,24]. Patients with axial disease experience varying effects on spinal radiographic progression as a result of biologic therapy [25]. In our cohort, the early detection of axial disease enabled the timely initiation of biologic therapy, potentially limiting radiographic progression.

In addition, patients with a family history of spondyloarthritis have a higher risk of developing both sacroiliitis and persistent active disease [26]. In our cohort, 17 (13.8%) JSpA patients had a SpA family history. Family history of SpA was shown to contribute significantly to specificity for the new AxJSpA criteria.

The criterion validity of the new classification system was compared to that of the ASAS axial SpA criteria [14], the ILAR ERA criteria [17], the ILAR ERA definition of sacroiliitis [11], the ASAS peripheral SpA criteria [15], and the European Spondyloarthropathy Study Group (ESSG) criteria [18] for adults. These results showed that the ESSG criteria had the highest sensitivity at 92% (113/123), while the new jAxSPA criteria had the lowest sensitivity at 61% (74/123). ASAS peripheral at 84% (62/74) had the highest specificity, followed by ASAS axial criteria with the lowest specificity.

In a study examining the performance of ILAR and ASAS classification criteria in ERA patients, the sensitivities of ILAR and ASAS criteria for axial SpA and peripheral SpA at diagnosis were 74.0%, 21.3%, and 85.1%, respectively. Specificities were 100%, 99.1%, and 90.9% at diagnosis. We did not differentiate ERA patients with axial involvement, and the control group differed in terms of quantity and content, leading to differences in specificity and sensitivity. We excluded patients with only peripheral involvement and enthesitis from our study, while the previous study, which used a different design, included psoriatic arthritis, oligoarticular JIA, and polyarticular JIA patients in its control group. Given the frequency of peripheral arthritis in our patient cohort, the inclusion of synovitis as an alternative entry criterion in the ESSG criteria may explain its high sensitivity, providing a wide range of options beyond the entry criteria. Similarly, in another study, the sensitivity and specificity of the ESSG criteria were high at 85.4% and 96.4%, respectively [27].

ASAS criteria for SpA validated in 2982 rheumatic children [28], revealed 78.7% for sensitivity and 92.2% for specificity. Sensitivity of the criterion “inflammatory back pain” was only 9.1% in children. The ASAS axial SpA criteria indicate that a history of back pain for a minimum of three months is necessary as an entry criterion prior to conducting MRI and/or radiographic investigations of the sacroiliac joints. In the absence of back pain, there seems to be no obvious clinical rationale for conducting MRI studies of the sacroiliac joints and spine in children. These may explain the low sensitivity of our study group to ASAS axial criteria. The additional criteria of uveitis, psoriasis, IBD, and the absence of a prior infection history in patients with peripheral arthritis within this criterion may account for the low sensitivity of our cohort to ASAS peripheral criteria.

Our study has several limitations. Our data are retrospective; not all data were available for certain time points. We used expert opinions as a gold standard. The duration of follow-up was heterogeneous. Our center performed MRI examinations based on the clinical judgement of the treating physician, primarily when there was lumbosacral pain. As discussed earlier, axial disease can be clinically silent. Our population was followed in a tertiary center, with recruitment likely biased towards more severe cases.

This is the first external validation of the newly proposed AxJSpA criteria using an independent pediatric cohort. In our cohort, the AxJSpA criteria demonstrated high specificity but relatively low sensitivity, likely influenced by the strong emphasis on structural MRI findings, which are often absent in early-stage or mild pediatric cases. Although reducing the weight of such imaging findings might improve sensitivity, this may increase misclassification in children with non-inflammatory back pain. These results underscore the need for prospective validation studies to refine the scoring thresholds and optimize diagnostic performance across diverse pediatric populations. Furthermore, we suggest that similar to the ASAS framework, a separate set of criteria could be considered for SpA patients with prominent peripheral involvement but without axial disease to ensure the inclusion of clinically relevant non-axial cases and improve cohort homogeneity.

## Figures and Tables

**Table 1:** Axial Juvenile Spondyloarthritis (AxJSpA) criteria weights and scoring [16].

Criteria Domains and Levels			Maximum	
Level			Weight	Domain Score
Imaging: active inflammation				
0	No unequivocal evidence of active lesions typical of sacroiliitis on MRI of the SIJs		0	
1	Unequivocal evidence of active lesions typical of sacroiliitis on MRI of the SIJs		23	23
Imaging: structural lesions				
0	No unequivocal evidence of structural lesions typical of sacroiliitis on MRI of the SIJs		0	
1	Unequivocal evidence of sacroiliitis on radiograph ^		13	
2	Unequivocal evidence of structural lesions typical of sacroiliitis on MRI of the SIJs		23	23
Pain chronicity				
0	No pain or pain < 4 days per week or for <6 weeks		0	
1	Most days (≥4 days/week) for ≥6 but <12 weeks		6	
2	Most days (≥4 days/week) ≥ 12 weeks		9	9
Pain pattern				
0	No identifiable pain pattern		0	
1	Awakens patient second half of the night OR insidious onset		6	
2	Moderate to total relief with non-steroidal anti-inflammatory drugs		10	
3	Improves with activity		13	13
Pain location				
0	No back, groin, hip, or buttock pain		0	
1	Lumbar spine pain (patient-reported)		6	
2	Sacroiliac pain with deep palpation/FABER/Mennell/Gaenslen maneuvers # OR groin/hip pain (patient-reported)		11	
3	Sacral/buttock pain (patient-reported)		12	12
Stiffness (morning)				
0	No stiffness or stiffness < 15 min		0	
1	≥15 min		9	9
Genetics				
0	No relevant family history in 1st degree relative and/or HLA-B27 unknown or negative		0	
1	Presence of the following in a 1st degree relative: SpA or HLA-B27-associated acute (symptomatic) anterior uveitis		8	
2	Presence of HLA-B27		11	11

The maximum AxJSpA score is 100, and scores of 55 and above meet the threshold for classification as AxJSpA. Levels within each domain are mutually exclusive. The highest level achieved within each domain contributes to the overall score. ^ Level only applicable in the absence of pelvic MRI. # FABER = Flexion Abduction External Rotation of the hip (SIJ pain should be on contralateral side); Mennell’s maneuver: application of SIJ pressure with the patient in prone position, leg straight, and hip extended; Gaenslen’s test: patient is supine with one hip and knee flexed, and the examiner applies downward force to hyperextend the other leg along the edge of the examining table (SIJ pain is on the ipsilateral side being hyperextended).

**Table 2:** Classification criteria for juvenile spondyloarthritis.

ILAR Classification Criteria for Enthesitis-Related Arthritis [17]	ASAS Classification Criteria for Peripheral Spondyloarthritis [15]	ASAS Classification Criteria for Axial Spondyloarthritis [13]	ESSG Criteria [18]
Inclusion criteria Arthritis and enthesitis OR Arthritis or enthesitis plus two of the following: Sacroiliac joint tenderness and/or inflammatory spinal pain HLA-B27 positivity Onset of arthritis in boys aged > 6 years Family history in at least one first-degree relative of ankylosing spondylitis, ERA, sacroiliitis with IBD, reactive arthritis, or acute anterior uveitis Acute anterior uveitis **Exclusion criteria** Psoriasis or a history of psoriasis in the patient or a first-degree relative Presence of IgM RF on at least two occasions at least 3 months apart Systemic JIA in the patient	Arthritis and/or enthesitis and/or dactylitis **plus** the following: ≥1 SpA feature Uveitis Psoriasis Inflammatory bowel disease Preceding infection HLA-B27 Sacroiliitis on imaging **OR** ≥2 other SpA features Arthritis Enthesitis Dactylitis Inflammatory back pain ever Positive family history of SpA	In patients with ≥3 months of back pain and age < 45 years Sacroiliitis on imaging plus ≥ 1 SpA feature **OR** HLA-B27 **plus** ≥2 other SpA features SpA features Inflammatory back pain Arthritis Enthesitis (heel) Uveitis Dactylitis Psoriasis Crohn’s/ulcerative colitis Good response to NSAIDs Family history of SpA HLA-B27 Elevated CRP	Inflammatory back pain or synovitis (asymmetric or predominantly in the lower limbs) **plus** ≥1 of the following: Positive family history Psoriasis Inflammatory bowel disease Urethritis or acute diarrhea occurring within 1 month before arthritis Alternating buttock pain Enthesopathy Radiologic evident sacroiliitis
**ILAR sacroiliac joint arthritis (pediatric):** Presence of tenderness upon direct compression over the sacroiliac joints [11].			

Abb: ASAS, Assessment of the SpondyloArthritis International Society; CRP, C-reactive protein; ERA, enthesitis-related arthritis; HLA, human leucocyte antigen; IBD, inflammatory bowel disease; IgM, immunoglobulin M; ILAR, International League of Associations for Rheumatology; JIA, juvenile idiopathic arthritis; NSAIDs, non-steroidal anti-inflammatory drugs; PRINTO, Pediatric Rheumatology International Trials Organization; RF, rheumatoid factor.

**Table 3:** Demographic and clinical characteristics of the patients.

	JSpA Patients (*n* = 123)	Control Group (*n* = 74)	*p* Value
Sex, female *n* (%)	32 (26%)	35 (47.3%)	0.002
Age at the time of diagnosis (mean ± SD years)	13 ± 2.8	10.7 ± 3.8	<0.001
Follow-up time (months)	98 ± 43	37.6 ± 38.1	<0.001
HLA-B27 presence *n* (%)	69 (56.1%)	4 (5.4%)	<0.001
Family history of SpA *n* (%)	17 (13.8%)	3 (4.1%)	0.028
Peripheral arthritis *n* (%)	80 (65%)	23 (31.1%)	<0.001
Enthesitis *n* (%)	59 (48%)	10 (13.5%)	<0.001
SIJ pain with direct palpation or FABER/Mennell/Gaenslen’s maneuvers * *n* (%)	79 (64.2%)	22 (29.7%)	<0.001
Acute anterior uveitis *n* (%)	4 (3.3%)	0	0.149
Inflammatory bowel disease *n* (%)	5 (4.1%)	3 (4.1%)	0.652
Psoriasis *n* (%)	0	3 (4.1%)	0.052
Active sacroiliitis (MRI) *n* (%)	78 (63.4%)	33 (44.5%)	0.010
Chronic sacroiliitis (MRI) *n* (%)	59 (47.9%)	14 (18.9%)	<0.001

* FABER = Flexion Abduction External Rotation of the hip (SIJ pain should be on the contralateral side); Mennell’s maneuver: application of SIJ pressure with the patient in prone position, leg straight, and hip extended; Gaenslen’s test: patient is supine with one hip and knee flexed, and the examiner applies downward force to hyperextend the other leg along the edge of the examining table (SIJ pain is on the ipsilateral side being hyperextended).

**Table 4:** Sensitivity, specificity, and negative and positive predictive values of different criteria for the classification of juvenile spondyloarthritis patients.

	Sensitivity (*n* = 123)	Specificity (*n* = 74)	Positive Predictive Value	Negative Predictive Value
jAxSPA	61% (74/123)	77% (57/74)	81.30%	53.70%
ASAS axial	74% (91/123)	65% (48/74)	77.80%	60.80%
ASAS peripheral	67% (83/123)	84% (62/74)	87.40%	60.80%
ESSG	92% (113/123)	68% (50/74)	82.50%	83.30%
ILAR	85% (105/123)	81% (60/74)	88.20%	76.90%
ILAR + SI	67% (82/123)	74% (55/74)	81.20%	57.30%

ASAS = Assessment of the SpondyloArthritis International Society, AUROC = Area Under the Receiver Operating Characteristic Curve, AxJSpA = axial juvenile spondyloarthritis, ERA = enthesitis-related arthritis, ESSG = European Spondyloarthropathy Study Group, ILAR = International League of Associations for Rheumatology, SpA = spondyloarthritis, NPV = negative predictive value, PPV = positive predictive value.

**Table 5:** Sensitivity and specificity of each criterion in juvenile spondyloarthritis patients.

Parameters of the AxJSpA Classification Criteria	Case Group (%)	Control Group (%)
Imaging: active inflammation	63.4	45
Imaging: structural lesions	48	20
Pain chronicity		
No pain or pain < 4 days per week or for <6 weeks	1.6	16.2
Most days (≥4 days/week) for ≥6 but <12 weeks	8.1	24.3
Most days (≥4 days/week) ≥ 12 weeks	90.2	59.5
Pain pattern		
No identifiable pain pattern	54.5	47.3
Awakens patient second half of the night OR insidious onset	4.1	12.2
Moderate to total relief with non-steroidal anti-inflammatory drugs	25.2	23
Improves with activity	16.3	17.6
Pain location		
Lumbar spine pain (patient-reported)	10.6	21.6
Sacroiliac pain with deep palpation/FABER/Mennell/Gaenslen maneuvers # OR groin/hip pain	1.6	6.8
Sacral/buttock pain (patient-reported)	78.9	41.9
Stiffness (morning)		
≥15 min	55.3	77
Genetics		
Presence of the following in a 1st degree relative: SpA or HLA-B27-associated acute anterior uveitis	4.1	2.7
Presence of HLA-B27	55.3	5.4

AxJSpA: axial juvenile spondyloarthritis; # FABER: Flexion Abduction External Rotation of the hip (SIJ pain should be on the contralateral side); Mennell’s maneuver: application of SIJ pressure with the patient in prone position, leg straight, and hip extended; Gaenslen’s test: patient is supine with one hip and knee flexed, and the examiner applies downward force to hyperextend the other leg along the edge of the examining table (SIJ pain is on the ipsilateral side being hyperextended).

## Data Availability

The data that support the findings of this study are available from the corresponding author upon reasonable request, subject to ethical restrictions.

## Supplementary Materials

The following supporting information can be downloaded at https://www.mdpi.com/article/10.3390/diagnostics15121498/s1, Figure S1: Annual Distribution of Juvenile Spondyloarthritis Diagnoses at Hacettepe University (2005–2024).
