# Supplementary material for: Validation of Axial Juvenile Spondyloarthropathy Criteria in Turkish Juvenile Spondyloarthropathy Patients
Source: Diagnostics (Basel). 2025 Jun 12;15(12):1498. doi: 10.3390/diagnostics15121498 (PMC12191953; doi:10.3390/diagnostics15121498)
Supplement: Supplementary file 1 [file diagnostics-15-01498-s001.zip › diagnostics-3501904-supplementary.pdf]

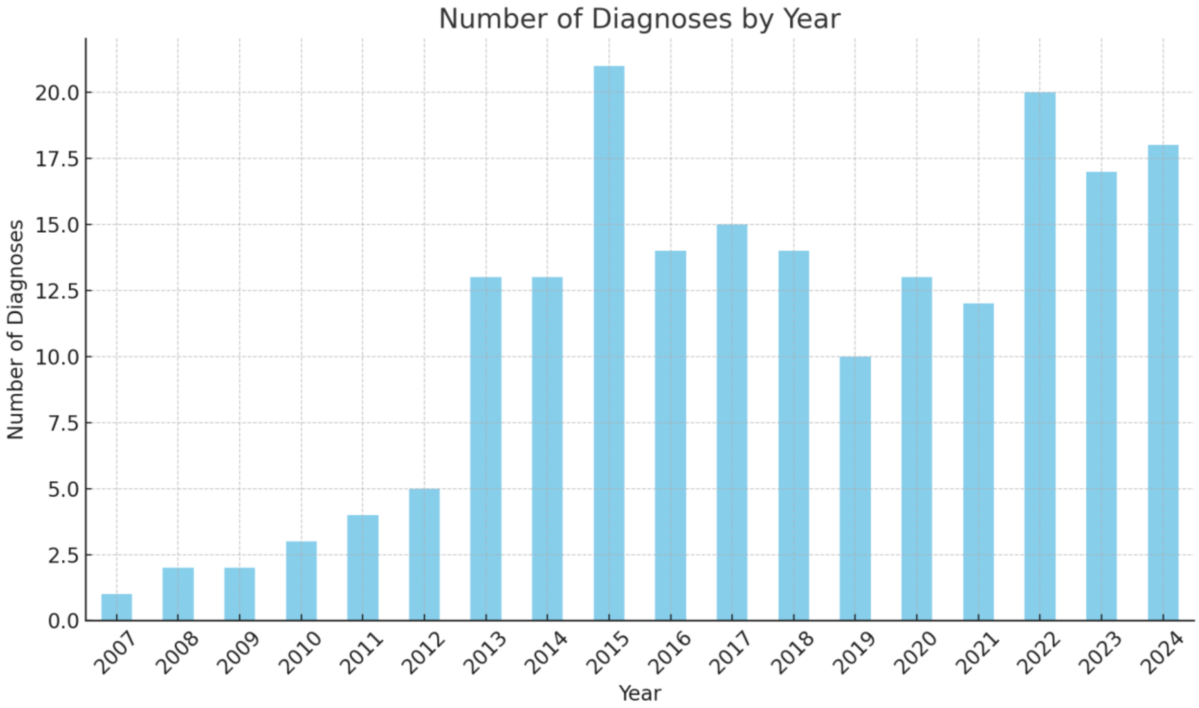

Supplementary Figure 1. Annual Distribution of Juvenile Spondyloarthritis Diagnoses at Hacettepe University (2005–2024)
